# Doxorubicin-Loaded Extracellular Vesicles Enhance Tumor Cell Death in Retinoblastoma

**DOI:** 10.3390/bioengineering9110671

**Published:** 2022-11-10

**Authors:** Wissam Farhat, Vincent Yeung, Francesca Kahale, Mohit Parekh, John Cortinas, Lin Chen, Amy E. Ross, Joseph B. Ciolino

**Affiliations:** 1 Department of Ophthalmology, Schepens Eye Research Institute of Mass Eye and Ear, Harvard Medical School, Boston, MA 02114, USA; 2 Division of Newborn Medicine, Department of Pediatrics, Boston Children’s Hospital, Harvard Medical School, Boston, MA 02114, USA; 3 Department of Ophthalmology, Affiliated Hospital of Zunyi Medical University, Zunyi 563000, China

**Keywords:** retinoblastoma, extracellular vesicles (EVs), targeted drug delivery, nanomedicine

## Abstract

Chemotherapy is often used to treat retinoblastoma; however, this treatment method has severe systemic adverse effects and inadequate therapeutic effectiveness. Extracellular vesicles (EVs) are important biological information carriers that mediate local and systemic cell-to-cell communication under healthy and pathological settings. These endogenous vesicles have been identified as important drug delivery vehicles for a variety of therapeutic payloads, including doxorubicin (Dox), with significant benefits over traditional techniques. In this work, EVs were employed as natural drug delivery nanoparticles to load Dox for targeted delivery to retinoblastoma human cell lines (Y-79). Two sub-types of EVs were produced from distinct breast cancer cell lines (4T1 and SKBR3) that express a marker that selectively interacts with retinoblastoma cells and were loaded with Dox, utilizing the cells’ endogenous loading machinery. In vitro, we observed that delivering Dox with both EVs increased cytotoxicity while dramatically lowering the dosage of the drug. Dox-loaded EVs, on the other hand, inhibited cancer cell growth by activating caspase-3/7. Direct interaction of EV membrane moieties with retinoblastoma cell surface receptors resulted in an effective drug delivery to cancer cells. Our findings emphasize the intriguing potential of EVs as optimum methods for delivering Dox to retinoblastoma.

## 1. Introduction

Retinoblastoma is the most prevalent pediatric intra-ocular malignancy, accounting for 10 to 15% percent of cancers occurring before the age of 5 [1,2,3]. Retinoblastoma can be sporadic or inherited and is mainly caused by the biallelic loss of tumor-suppressor gene RB1, conferring a rapid proliferative capacity to tumor cells [4,5]. Retinoblastoma is potentially fatal if left untreated, in which about 70% of patients in low- and middle-income countries die from this disease, compared with 3–5% in developed and high-income countries [6].

An array of treatment options for retinoblastoma is available, including systemic or intraarterial chemotherapy, cryotherapy, laser photoablation, radioactive plaque, and enucleation [7,8]. The optimal treatment method is based on tumor location, size, laterality, vitreous seed, and vision prognosis. Intra-arterial chemotherapy delivery through the ophthalmic artery has gained popularity in the last decade in efforts to deliver high doses of localized chemotherapy while avoiding systemic side effects [9,10,11]. However, the currently available treatment options typically result in substantial adverse effects, including facial abnormalities, cataracts, radiation retinopathy, and an increased risk of subsequent cancers. Efficacy of systemic chemotherapy, for example, is limited by systemic toxicity, rapid systemic clearance, and multidrug resistance [12,13,14]. In addition, the delivery of systemic medications to the retina is limited by the blood–retina barrier [15]. As a component of the central nervous system, the blood–retina barrier contains tight junctions, which help protect the retina from infections and aid in the maintenance of glycolysis for the tissue that has a high oxygen consumption rate [16,17]. The presence of these junctions could limit systemic drug delivery to the retina. To overcome these limitations, drug delivery by targeted therapy (use of drugs and substances to precisely identify and attack cancer cells via targeting cancer-specific markers, e.g., overexpressed genes and proteins) is known to have several advantages over systematic strategies: fewer doses are needed, toxicity and bioavailability can be better controlled, release durations can be extended, local and systemic drugs can be combined with different kinetics, a controlled release directly to the site of retinoblastoma, and systemic drug exposure can be avoided. Targeting retinoblastoma via safe and effective means would be beneficial to most patients with the disease, particularly in developing nations, where survival rates are low due to impoverished educational, socioeconomical, and healthcare systems.

Approaches for targeted delivery of therapeutics in cancer (e.g., retinoblastoma) may involve nanomedicine, which focuses on the development of nanocarriers for preventative, diagnostic, and therapeutic purposes. Nanocarriers are nanoparticles (1–100 nm in diameter) that have been used in a variety of therapeutic applications, including the targeted delivery of anticancer drugs into tumors [18]. These nanosized particles have demonstrated several benefits in cancer care, including improved pharmacokinetics, selective targeting, minimized toxic side effects, and reduced drug resistance [19,20,21].

Extracellular vesicles (EVs) are nanosized vesicles, composed of a lipid bilayer, and contain mRNA, miRNA, lipids, and proteins reflective of the original cell type [22,23]. A range of biological functions has been proposed for EVs, such as removing superfluous or harmful cellular contents, releasing signaling molecules, modifying cell functions, propagating pathogens, stimulating or inhibiting the immune system, and presenting antigens, among others [24,25,26]. Given their endogenous targeting ability and physiochemical stability, EVs as disease markers and drug cargos emerged as a promising field in biomedical research [27,28]. Their potential role, particularly for targeted therapy delivery, is being studied in various disorders affecting the immune, nervous, cardiovascular, endocrine, and hepatobiliary systems [29,30,31,32,33,34,35].

EVs as diagnostic modalities and treatment tools are being used for a variety of ocular diseases [36,37]. In patients with uveal melanoma, EVs isolated from serum and other tissues depicted miRNA alterations, aiding in differentiation from healthy controls [38,39]. Regulatory T-cell-derived EVs conferred immune suppression following corneal transplantation, leading to lower rates of rejection [40]. EVs derived from corneal myofibroblasts were shown to increase corneal epithelial cell proliferation [41]. The therapeutic potential of EVs has gained a lot of interest, specifically in chronic retinal pathologies, such as diabetic retinopathy and macular degeneration [42,43].

In this study, we isolated EVs from two metastatic breast cancer cell lines (4T1 and SKBR3) that express a marker that selectively interacts with retinoblastoma cells and characterized their molecular and biophysical properties in compliance with guidelines from the International Society of Extracellular Vesicles (ISEV) [44]. Doxorubicin hydrochloride (Dox) with different concentrations was loaded into cell lines (4T1 and SKBR3) before EV isolation using the endogenous loading machinery of the cells, resulting in subsequent EV loading with Dox. Dox was selected as a model drug, as it is a broadly used chemotherapeutic agent for the treatment of retinoblastoma. Dox-loaded EVs were then isolated and the drug content of EVs was measured. Y79 human retinoblastoma cells were then incubated with EVs derived from both cells and free Dox as a control group and the difference in cell death was compared. Our study demonstrated (1) a higher incorporation of drug in 4T1 cells derived EVs and (2) a higher amount of apoptotic retinoblastoma cells in groups incubated with EVs loaded with Dox as compared to free drug. Overall, the findings of this study demonstrated that locally delivered Dox-loaded EVs have the potential to treat retinoblastoma while avoiding systemic side effects.

## 2. Materials and Methods

### 2.1. Cell Culture

Mouse 4T1 mammary carcinoma cells (CRL-2539™, ATCC, Manassas, VA, USA) and human SKBR3 breast cancer cells (HTB-30™, ATCC) were cultured in Dulbecco’s modified Eagle’s medium (DMEM, Gibco, ATCC formulated) supplemented with 10% fetal bovine serum (FBS), 100 mg/mL streptomycin, 100 U/mL penicillin, and 1 mM L-glutamine. Cells were grown at 37 °C in a humidified 95% air and 5% CO_2_ atmosphere. Human Y79 retinoblastoma cells (HTB-18™, ATCC) were cultured in Roswell Park Memorial Institute 1640 medium (RPMI 1640, Gibco, ATCC formulated) supplemented with 10% FBS, 100 mg/mL streptomycin, 100 U/mL penicillin, and 1 mM L-glutamine. Cells were grown at 37 °C in a humidified 95% air and 5% CO_2_ atmosphere.

### 2.2. Doxorubicin Loading and Extracellular Vesicle (EV) Isolation

The loading of doxorubicin into the EVs was performed by loading the cells before EV isolation. In this approach cells from which the EVs are derived are incubated with different concentrations of doxorubicin and the endogenous loading machinery of the cells resulted in subsequent EV loading. Separation and characterization of doxorubicin-loaded EVs were in accordance with the minimum information for studies of extracellular vesicles (MISEV) [44], as summarized by ISEV. EVs are characterized by their expression of vesicle-associated proteins (CD9, CD63, CD81, and ALIX). In this investigation, breast cancer cells (4T1 and SKBR3) were grown for 2 days in serum-free medium with varying doxorubicin doses (0 M, 1 M, 10 M, and 50 M), the conditioned media (CM) were collected, and EVs were extracted, as previously described [45]. In short, breast cancer CM was subjected to successive differential centrifugation, to remove cells (300× *g* for 10 min), cellular debris (3000× *g*, for 10 min), and apoptotic detritus (13,000× *g* for 30 min). The supernatant was concentrated using a Centricon^®^ Plus-70 centrifugal filter unit with a 100 kDa MW cutoff (MilliporeSigma, Burlington, MA, USA) and ultra-centrifuged for 1 h and 10 min at 110,000× *g* (4 °C) using a Beckman Type 50.2 Ti Rotor (Beckman Coulter, Brea, CA, USA) in a Beckman Coulter, Optima 140 LE-80K Ultracentrifuge. The resultant pellet was resuspended in phosphate-buffered saline (PBS: Gibco), centrifuged again for 1 h and 10 min at 110,000× *g* (4 °C), and stored at −80 °C until further use.

### 2.3. Western Blot

Protein isolation and Western blot were carried out in the same manner as previously described [46,47,48]. Proteins from EVs were extracted with RIPA buffer (10 mM Tris, 150 nM NaCl, 1% deoxycholic acid, 1% Triton X, 0.1% SDS, and 1 mM EDTA) containing protease inhibitors (aprotinin, PMSF, and sodium orthovanadate). A Pierce^TM^ bicinchoninic acid (BCA) protein assay kit was used to assess protein content (Thermo Fisher Scientific, Waltham, MA, USA). A 4–20% Tris-Glycine gel (Bio-Rad, Hercules, CA, USA) was loaded with equal quantities of protein (20 g/lane) and electrophoresed under nonreducing conditions. Proteins were transferred to a PVDF membrane, blocked for 2 h in blocking buffer (PBS, 0.05 percent Tween20^®^, 5% milk), and then probed overnight with the following primary antibodies: ALIX (Sc-166952; Santa Cruz, CA, USA); CD81 (Sc-7637; Santa Cruz); and GM130 (#12480; Cell Signaling, Danvers, MA, USA). The membranes were rinsed the next day and treated for 1 h at room temperature (RT) with the secondary antibodies donkey anti-mouse IRDye 800CW and donkey anti-rabbit IRDye 680RD (1:2000, LI-COR Biosciences, Lincoln, NE, USA). All antibodies were diluted according to the manufacturer’s instructions. A fluorescence scanner was used to photograph the membranes (Odyssey v.3.0, LI-COR Biosciences).

### 2.4. TRIFIc Exosome Assay

CD9 and CD63 were directly analyzed using TRIFic exosome assays (CD9: Cat. No.: EX501, Cell Guidance Systems Ltd., Cambridge, UK) and (CD63: Cat. No.: EX502, Cell Guidance Systems Ltd., Cambridge, UK), respectively, by following the manufacturer’s guidelines. Briefly, streptavidin-coated plates were incubated with biotinylated Anti-CD9 or Anti-CD63 for 1 h at room temperature. The supernatant was removed and plates were washed three times with the provided buffer. The EV samples were transferred to the wells, incubated for 1 h, and washed three times. 100 μL of Europium-labeled CD9 or CD63 antibodies was added to the wells for 1 h, all at room temperature. After another wash step, 100 μL enhancement solution was added and incubated for 15 min at room temperature. Europium time-resolved fluorescence was subsequently measured at 615 nm wavelength by a Synergy H1 Hybrid Multi-Mode Reader (Agilent Technologies, Santa Clara, CA, USA). Results were expressed as relative fluorescence units (RFUs). For each condition, all experiments were carried out in triplicate.

### 2.5. Nanoparticle Tracking Analysis (NTA)

All EV samples were produced and analyzed in the same manner as previously described [49,50]. PBS was used to dilute the EV samples to a final volume of 1 mL. Pretesting the Particle Metrix ZetaView^®^Basic NTA PMX-120 machine (Particle Metrix, Ammersee, Germany) at the optimal particle-per-frame value (140–200 particles/frame) yielded perfect measurement concentrations. For EVs/nanospheres, the manufacturer’s default software settings were used. Three cycles of scanning 11 cell locations and collecting 30 frames per position were conducted for each measurement under the following conditions: focus—autofocus; camera sensitivity for all samples—75; shutter—100; scattering intensity—detected automatically; cell temperature—25 °C. Following capture, the video was examined using the built-in ZetaView Software 8.04.02 SP2 with the following parameters: maximum area—1000; minimum area—5; minimum brightness—25; hardware—embedded laser, 40 mW at 488 nm; camera—CMOS. In order to reduce data skewing caused by single big particles, the number of completed tracks in NTA measurements was always more than the specified minimum of 1000 [51].

### 2.6. Transmission Electron Microscopy (TEM)

As previously disclosed, EVs were fixed and photographed using transmission electron microscopy (TEM) to examine their shape [52]. In brief, the EV pellet was resuspended in 4 percent *w*/*v* paraformaldehyde (PFA) in PBS (Gibco) and fixed for 30 min at room temperature. Next, 5 µL of the fixed EV solution was placed in a formvar/carbon-coated grid (Electron Microscopy Sciences, Hatfield PA, CA, USA) and incubated for 20 min to allow the EVs to cling to the grid surface. The grids were rinsed with drops of PBS to eliminate residual PFA before being resuspended in 1 percent *v*/*v* glutaraldehyde in PBS for 5 min. By gently resuspending the grid in water, residual glutaraldehyde was eliminated. The grids were immersed in a uranyl–oxalate solution for 10 min before being incubated in a methylcellulose solution for contrast. Prior to TEM inspection, grids containing adsorbed EVs were dried (JEM-1220 TEM: JEOLUSA, Peabody, MA, USA).

### 2.7. Quantification of Doxorubicin

Doxorubicin was analyzed using a fluorometer, a Synergy H1 Hybrid Multi-Mode Reader (Agilent Technologies), and the concentrations were determined in relation to a standard curve starting from 50 µM to 500 µM and blanks of ultrapure water. The samples were excited at 488 nm and read at 530 nm. Effective loading was calculated as a function of µM doxorubicin per EV.

### 2.8. Annexin V Assay

Apoptosis was determined using an annexin V-based fluorescent assay (RealTime-Glo™ MT Cell Viability Assay, Promega, Madison, WI, USA), according to the manufacturer’s instructions. Under typical cell culture conditions, Y79 human retinoblastoma cells were plated in 96-well plates and grown to about 80% confluence. The medium was then removed and cells were washed twice with PBS. Next, a fresh medium containing 5 µg/mL Dox or Dox-loaded EVs generated from 4T1 or SKBR3 was added and incubated for 24 h. Luminescence was recorded using a Synergy H1 Hybrid Multi-Mode Reader (Agilent Technologies). Results were expressed as relative luminescence units (RLUs; apoptosis). For each condition, all experiments were carried out in triplicate.

### 2.9. Caspase Activity

CellEvent^TM^ Caspase-3/7 detection reagent was used to examine caspase activation (C10423, ThermoFisher). Under typical cell culture conditions, Y79 human retinoblastoma cells were plated in 96-well plates and grown to about 80% confluence. The cells were subsequently exposed to free Dox or Dox-loaded EVs generated from 4T1 or SKBR3 for 24 h. The media were then taken out and the reagent was given to the cells after being diluted in PBS (90 µL/mL). Fluorescence was measured using a Synergy H1 Hybrid Multi-Mode Reader (Agilent Technologies) after 10 min of incubation. The parameters for the excitation and emission were 500 and 530 nm, respectively. Results were expressed as relative fluorescence units (RFUs). For each condition, all experiments were carried out in triplicate.

### 2.10. EV Labeling with PKH26

Isolated EVs were fluorescently labeled with a PKH26 Red Fluorescent Cell Linker Kit (MilliporeSigma) according to the manufacturer’s instructions [45]. The EV pellet was resuspended in diluent C, incubated with PKH26 dye in diluent C buffer at a ratio of 1:1 for 2 min at RT, and mixed with bovine serum albumin (BSA, 1% *w*/*v* in diluent C; Sigma Aldrich, St. Louis, MO, USA) at an equal ratio per volume. The PKH26–EV solution was subjected to ultracentrifugation using a Beckman Type 50.2 Ti Rotor (Beckman Coulter) in an Optima LE-80K Ultracentrifuge (Beckman Coulter) at 110,000× *g* for 1 h and 10 min at 4 °C. The supernatant was removed and the PKH26-labeled EVs were washed, resuspended in diluent C, and ultracentrifuged (110,000× *g* for 1 h and 10 min at 4 °C). The wash/ultracentrifugation steps were repeated a total of three times. PKH26-labeled EVs were filter sterilized (0.22 μm pore) prior to use in cell culture. For the control sample, particle-free PBS (Gibco) was used instead of EVs and stained according to the procedures described above.

### 2.11. Cell–EV Interaction: Cellular Uptake of EVs

The Y79 cells were cultured in a 12-well plate up to 70% confluence. Following the incubation of cells at 37 °C and 5% CO_2_ for 24 h, the cells were treated with 4T1 or SKBR3-derived EVs or epithelial cell-derived EVs that were labeled with PKH26 for another 24 h. At the end of the culture period, the cells along with the media were collected in the flow cytometry tube directly as the cells are non-adherent in nature. The cells were centrifuged at 148× *g* for 5 min. Following centrifugation, the supernatant was discarded and the cells were washed with PBS three times to remove any excess stains or media remnants. The cells were then re-centrifuged at the same settings and re-suspended in 200 μL of PBS for analysis. Flow cytometry was performed using BDLSRII machine. At least 10,000 events were recorded per sample with a maximum flow rate of 500 events/s. PKH26-derived fluorescence intensity is obtained using the FITC channel. All the experiments were performed in triplicate for each condition.

### 2.12. Statistics

Data were reported as Mean ± SEM unless stated otherwise. Differences between groups were compared by ANOVA followed by Tukey’s multiple-comparison test. *p* values < 0.05 were considered significant * *p* < 0.05, ** *p* < 0.01, and *** *p* < 0.001.

## 3. Results

### 3.1. Molecular Characterization of Dox-Loaded EVs

To understand whether the inclusion of Dox treatment influenced the molecular properties of 4T1- and SKBR3-derived EVs, we characterized the expression of EV-associated markers. We employed an established EV isolation methodology [45,53] to determine if EVs were effectively extracted from 4T1 and SKBR3. EVs were characterized using ISEV standards by identifying proteins expressed in EVs and by determining their biophysical characteristics [54]. EVs in our investigation were adjusted to protein content.

**Figure 1:**
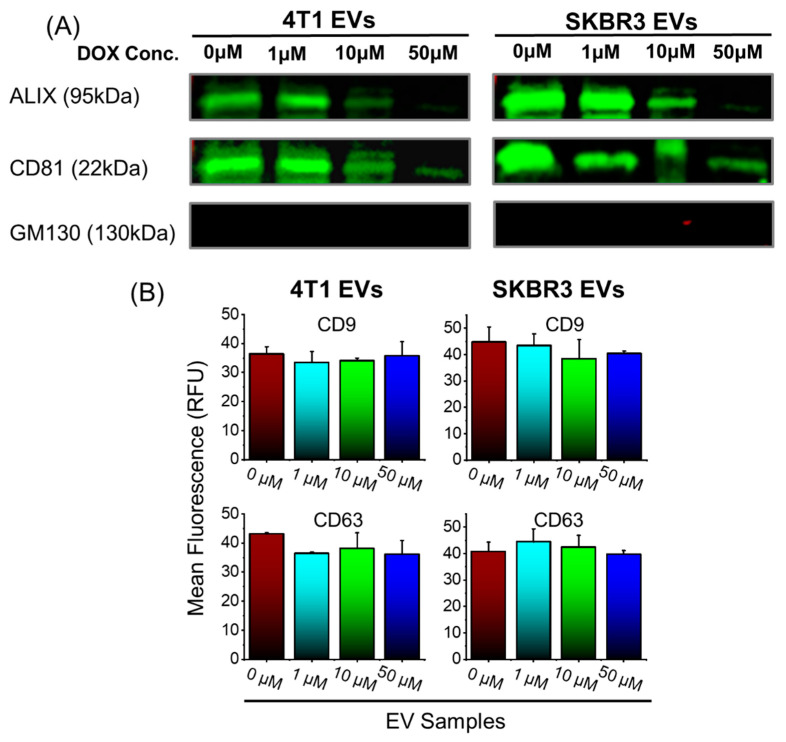
Isolation and molecular EV characterization of 4T1 and SKBR3 EVs loaded with Dox. (**A**) EV pellets (20 μg protein/lane) were examined by Western blot to probe for EV-associated markers ALIX, CD81, and negative control (GM130). Representative images are shown for vesicle-associated proteins. (**B**) EV pellets were examined by TRIFIc exosome assay to probe for vesicle-associated markers CD9 and CD63. Data are presented as mean + SEM for three independent EV preparations.

We cultured 4T1 and SKBR3 for 2 days in serum-free media and then added increasing concentrations of doxorubicin (0 µM, 1 µM, 10 µM, and 50 µM) to determine whether efficacy or toxicity was dose dependent. These concentrations were found optimal and did not induce significant cell death in 4T1 or SKBR3. The conditioned media were collected and EVs were isolated, as previously described [45]. We used Western blotting and TRIFic exosome assays to examine vesicle-associated markers ALIX, CD9, CD63, and CD81 (Figure 1A,B). The EVs derived from 4T1- and SKBR3-treated cells (treated with 0 µM and 1 µM Dox) had comparable expression levels of ALIX and CD81 (Figure 1A), as well as CD9 and CD63 (Figure 1B). With a Dox concentration of 10 µM, there was a modest reduction in ALIX and CD81 expression (Figure 1A) from 4T1- and SKBR3-derived EVs. 4T1 and SKBR3 EV expression of ALIX and CD81 was reduced drastically with 50 µM Dox treatment. There were no significant differences in CD9 and CD63 expression with increasing Dox concentration (Figure 1B). The non-EV protein GM130 expression of all EV preparations was negative, indicating there were no contaminants present.

### 3.2. Biophysical Characterization of Dox-Loaded EVs

**Figure 2:**
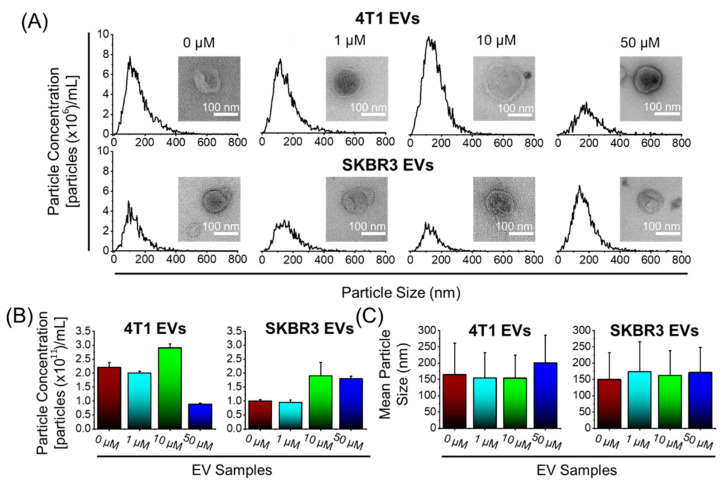
Biophysical characterization of 4T1 and SKBR3 EVs loaded with Dox. (**A**) Nanoparticle tracking analysis (NTA) was performed on EV pellets and a size distribution histogram for each EV sample is displayed. Transmission electron microscopy pictures of EV morphology (scale bar = 100 nm) ((**A**), inset). (**B**) NTA with Zetaview^TM^ was used to quantify the average particle concentrations (particles ×10^11^/mL) and (**C**) mean particle size (nm) of EV pellets. Data are presented as mean + SEM for three different EV preparations.

To confirm whether the inclusion of Dox treatment influenced the biophysical features of the EVs, we conducted NTA analyses to investigate the particle size distribution (Figure 2) and the EV morphological features by TEM (Figure 2A, inset). We show that NTA size distribution profile of the EVs is not affected by the Dox treatment dose. In addition, TEM showed that all EVs were approximately spherical in shape and contained lipid bilayers. Furthermore, TEM images showed that the morphology of EVs remained unchanged after Dox loading. There were no significant variations in particle concentration measurements of EVs ranging from 0.9 × 10^11^ to 2.9 × 10^11^ particles/mL (Figure 2B); mean particle size measurements ranging from 149 to 200 nm (Figure 2C); and a net negative surface charge (data not shown), indicating colloidal EV stability. Overall, the findings revealed that Dox treatment had no influence on the biophysical features of the EVs.

### 3.3. Assessing the Quantity of Dox Loaded into EV

**Figure 3:**
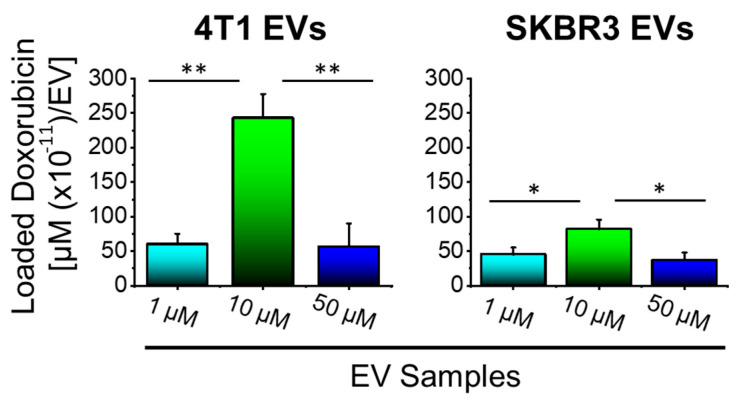
10 µM Dox enhances drug loading into 4T1 and SKBR3 cell-derived EVs. An effective loading of EVs was obtained when cells were treated with 10 µM Dox compared to 1 μM or 50 μM. 4T1 EVs showed a higher Dox loading compared to SKBR3 EVs. Data are presented as mean + SEM for three independent preparations. Significance calculated by ANOVA and is illustrated as follows: * *p* < 0.05; ** *p* < 0.01.

We performed fluorometry to quantify levels of Dox loaded in EVs. Dox-loaded EVs were isolated and the drug content of EVs was quantified by fluorometry and the effective loading calculated as a function of µM of Dox per EV in each volume (Figure 3), obtained by NTA. Results indicated higher incorporation of Dox in 4T1 EVs compared to SKBR3 EVs and that the treatment of 4T1 and SKB3 cells with 10 µM Dox led to the highest loading of about 243 × 10^−11^ and 82 × 10^−11^ µM Dox/EV, respectively. The increased concentration of Dox from 1 to 10 µM significantly increased drug incorporation into EV nanocarriers derived from both cell types. In contrast, the further increase in the Dox concentration from 10 to 50 µM resulted in a significant reduction in drug loading in both EV types. This is attributed to increased cell death due to the higher Dox dose (50 µM) and, hence, resulted in less drug loading. The usage of 10 µM Dox in treated cells resulted in the generation of EVs with the highest Dox-loading capacity. Therefore, 10 μM Dox EVs were used for all further investigations.

### 3.4. Dox-Loaded EVs Enhanced Retinoblastoma Cell Death In Vitro

**Figure 4:**
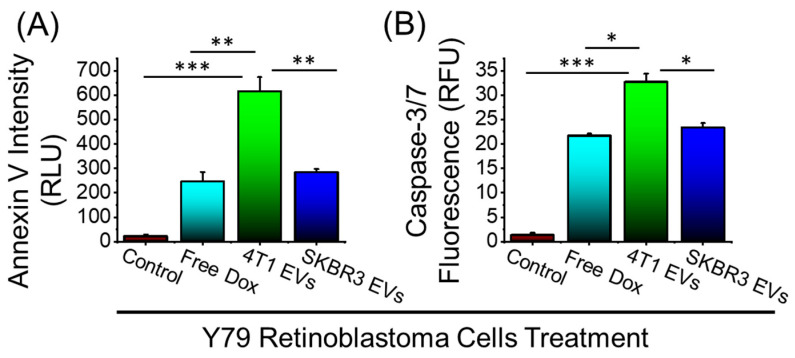
Anticancer efficacy of free Dox and Dox-loaded EVs in Y-79 human retinoblastoma cells. (**A**) Annexin V staining for detecting cell apoptosis after 24h treatment. (**B**) CellEvent^TM^ caspase-3/7 detection reagent after 24 h treatment. Data are presented as mean + SEM for three independent experiments. Significance calculated by ANOVA and is illustrated as follows: * *p* < 0.05; ** *p* < 0.01; *** *p* < 0.001.

Routinely, Dox has been used to treat retinoblastoma and, here, we assessed whether we could enhance the antitumor effect by using Dox-loaded EVs to treat retinoblastoma in vitro. The in vitro antitumor efficacy of Dox-loaded EVs against Y79 human retinoblastoma cells was assessed. Further, 5 µg/mL Dox or Dox-loaded EVs generated from 4T1 or SKBR3 was added to Y79 cells and incubated for 24 h. The degree of apoptosis was evaluated using Annexin V staining of externalized phosphatidylserine as a common method for detecting programmed cell death. As shown in Figure 4A, both types of Dox-loaded EVs enhanced the apoptotic levels of Y79 cells as compared to free Dox, demonstrating a greater effect of Dox-loaded EVs over free Dox. Furthermore, Y79 cell death demonstrated significantly enhanced cell death upon treatment with 4T1 EVs compared to SKBR3 EVs, attributed to the higher Dox loading, as observed in Figure 3. Although free Dox increased apoptosis and markedly inhibited growth when compared to the control, it was apparent that Dox-loaded 4T1 EVs showed greater levels of apoptotic activity than the free drug. To validate these findings, caspase 3/7 activation was evaluated (Figure 4B) since caspase 3/7 is uniformly activated during apoptosis. In agreement with Annexin V staining results, Dox and Dox-loaded EVs induced significant caspase 3/7 activation in Y79 cells. Dox-loaded 4T1 EVs showed an elevated level of caspase 3/7 activation compared to free Dox treatment alone. Overall, Dox-Loaded 4T1 EVs enhanced apoptotic activity that is indicative of inducing tumor cell death in Y79 cells compared to free Dox treatment.

### 3.5. Cell–EV Interaction

We next assessed whether the distinct molecular composition of 4T1 or SKBR3 EVs lead to enhanced delivery of Dox into target cells. Because 4T1 and SKBR3 EVs could show altered adhesion proteins and overexpressed surface receptors that can alter their cellular uptake, comparisons were made with a non-cancerous epithelial-cell-derived EV to determine their endocytic capacity.

**Figure 5:**
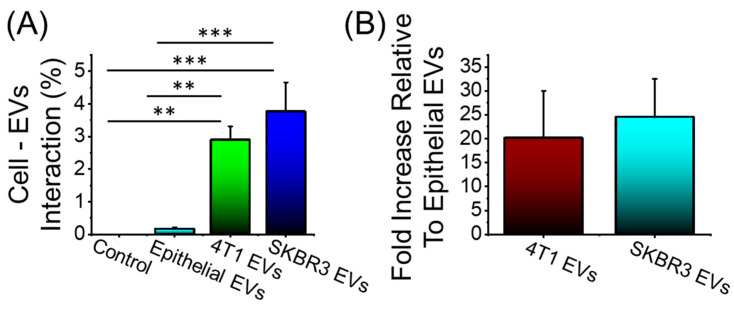
Cellular uptake of PKH26-labeled EVs after 24 h incubation time. (**A**) 4T1 EVs and SKBR3 EVs show a significant accumulation in Y79 cells compared to noncancerous epithelial EVs. (**B**) Fold increase in cancerous EV accumulation in Y79 cells relative to noncancerous EVs. No significant difference in the accumulation among the cancerous EV formulations. Data are presented as mean + SEM for three independent preparations. Significance calculated by ANOVA and is illustrated as follows: ** *p* < 0.01; *** *p* < 0.001.

Here, we examined the accumulation of EVs derived from breast cancer cells (4T1 and SKBR3) in Y79 cells against EVs derived from non-cancerous epithelial cells. Y79 cells were treated with different PKH26-labeled EVs for 24 h and the accumulation of the EVs was reported by flow cytometry (Figure 5A,B). Y79 cells accumulated elevated levels of fluorescently labeled 4T1 EVs and SKBR3 EVs compared to non-cancerous epithelial EVs (>20-fold increase, Figure 5B). Y79 uptake of cancerous EVs is extremely effective compared to noncancerous EVs, which could be due to altered cellular adhesion and potentially explains the efficacy compared to free drugs.

## 4. Discussion

The treatment modalities of retinoblastoma remain a complex process involving a multitude of different treatments that include a combination of chemotherapy, cryotherapy, immunotherapy, radiotherapy, and surgery, yet these are often met with severe side effects due to their non-specific targeting on healthy cells [55]. There has been great progress in creating novel methods for different therapeutic approaches, such as 3D bioprinting, hydrogels, and nanoparticles in different diseases [56,57,58,59,60]. Yet, within the past decade, EVs have emerged as a therapeutic tool and potential vehicle for targeted therapy due to their exceptional biocompatibility and physiochemical stability [36,61,62]. EVs are nanosized particles with a lipid bilayer that contains a repertoire of intrinsic cargo: mRNA, miRNA, lipids, and proteins derived from the origin cell type [63]. In recent years, studies have found that EVs contribute to the paracrine communication pathways, allowing for signaling cues to travel towards their specific targeted cell or tissue. More specifically, in the context of cancer (and retinoblastoma), EVs play an important role in tumor progression as messengers in cellular interactions and homeostasis of the tumor microenvironment. For instance, studies have shown that cancer EVs with specific surface markers, such as integrins [64,65] or proteoglycans [66,67], can enhance the endocytic uptake of EVs within tumor cells. Hence, the unique characteristics of cancer EVs make them a promising drug delivery system because they could be loaded with chemotherapeutics (with increased endocytic delivery to tumors) and used as a novel cancer treatment; this remains unexplored to date in retinoblastoma.

**Figure 6:**
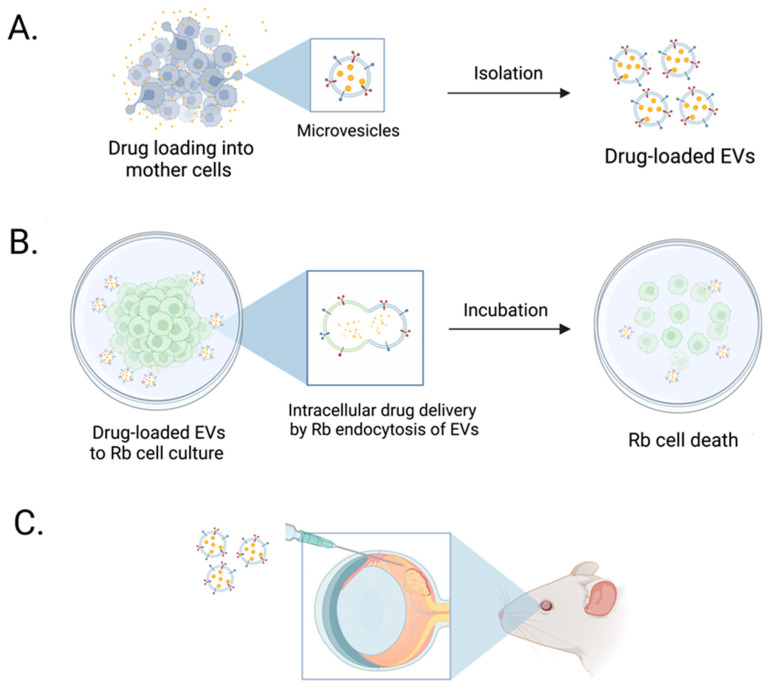
Schematic illustration of Dox-loaded EVs enhancing in vitro retinoblastoma cell death. (**A**) 4T1 and SKBR3 Dox-loaded EVs were isolated and characterized for the quantity of loaded Dox. (**B**) Dox-loaded EVs were added to retinoblastoma cell cultures in vitro and enhanced tumor cell death in Dox-loaded EVs compared to freely added drug. (**C**) Future studies will focus on assessing targeted drug delivery of Dox-loaded EVs in retinoblastoma tumor in in vivo models.

In this study, we first showed that we could load Dox into breast cancer cell lines (4T1 and SKBR3) at various doses utilizing the cells’ endogenous loading machinery; this resulted in isolating EVs with different loaded Dox concentrations (Figure 6). Next, we isolated and characterized EVs in accordance with the ISEV specifications [44]. Dox-loaded EVs (4T1- and SKBR3 EVs) were isolated and analyzed for their biophysical and molecular characteristics. We demonstrated that levels of tetraspanins CD9 and CD63 were present in all EV types [68,69], with decreasing levels of CD81 and ALIX in EVs with increasing Dox dosing levels. This decrease in ALIX and CD81 in 4T1 and SKBR3 EVs could be attributed to a high Dox dose (50µM) that leads to increased cell death (prior to Dox loading) and reduced secreted EVs, as summarized in numerous studies [70]. Moreover, our EV preparations were negative for levels of GM130, indicating that there were no impurities. Despite differences in their protein expression, we found an overlap in their biophysical characteristics when evaluating EV morphology, size distribution profiles, particle concentration, and zeta potential. Our data show an enrichment of EVs with an average size ranging from 149 to 200 nm and negative zeta potentials of all EVs. This suggests the existence of negatively charged phospholipids in the EV membrane, which contains major lipid species (glycosphingolipids, sphingomyelin, cholesterol, and phosphatidylserine) [71,72,73,74]. These findings indicate that we can successfully load Dox into EVs while retaining the biophysical and molecular characteristics of EVs in accordance with ISEV guidelines.

To test the functional efficacy of EVs as a drug delivery system, we studied the cytotoxicity of Y79 retinoblastoma cells treated with free Dox or Dox-loaded EVs, to determine whether Dox release via EVs enhances tumor cell death. Dox-loaded EVs greatly enhanced apoptotic activity (elevated caspase-3 and caspase-7) in Y79 cell in vitro compared to free Dox alone. The exact reason(s) why Dox-loaded EVs increased tumor cell death more than free Dox is not clear. It is possible that Dox-loaded EVs provide sustained release of the chemotherapeutic drug over a longer period compared to that of a single bolus of free Dox. Supporting this hypothesis are previous reports that slow release of Dox increases anti-cancer activity and decreases side effects [75,76,77,78]. In addition, it is possible that EVs form a drug depot in which the treatment modality is released slowly compared to the free drug, resulting in more effective exposure and sustained release of the drug. This slow drug release could be due to the shielding effect of the membrane envelope that prevents Dox from being destroyed and degraded by the harsh extracellular environment [79], enabling drug-loaded EVs to be more efficacious than free medicines. In fact, free Dox in solution is susceptible to a higher chance of deterioration and reduces its capacity to exert its anti-proliferative effects. Collectively, these are possible explanations as to why Dox-loaded EVs displayed elevated apoptotic activity in Y79 cells than free Dox at the same chemotherapeutic dose.

Multiple cytotoxicity studies were carried out and we found that Dox-loaded EVs caused more tumor cell death than free Dox. EVs have been reported to exhibit strong cellular endocytic activity, which may explain their potential for drug delivery to Y79 cells. To investigate this concept, the endocytic capacity of EVs by Y79 cells was tested in vitro to demonstrate the potential of 4T1 and SKBR3 EVs to enter retinoblastoma cells. In Y79 cells, we found that a substantially higher proportion of breast cancer EVs were endocytosed than non-cancerous ocular epithelial EVs. We propose that the accumulation of breast cancer EVs is related to the molecular composition of surface markers [64,65], which increases their capacity to become endocytosed compared to non-cancerous EVs. EV membrane proteins could include tetraspanins, integrins, or heparin sulfate proteoglycans; these may trigger increased cellular uptake by Y79 cells and mediate changes in signaling cascades [80,81,82]. There is also evidence that breast cancer cells that express specific markers would produce EVs [83,84,85] that can be internalized by Y79 cells via ligand-specific internalization, enhancing internalization and leading to increased cytotoxicity, which explains the efficiency of our Dox-loaded EV model. A culmination of different studies has reported the importance of membrane EV proteins in internalizing intrinsic EV cargo by targeted cells since they govern various tumor processes within the tumor microenvironment, including apoptosis, growth, inflammation, angiogenesis, invasion, and metastasis [86,87,88,89,90,91]. It is possible to hypothesize that breast cancer EVs are primed to target tumor cells and that the expression of key proteins could effectively deliver drugs that involve a combination of cell–EV receptor contact, internalization, and intracellular drug delivery, which we hope to use for novel retinoblastoma therapeutic approaches.

The scarcity of retinoblastoma-derived EVs that target their own microenvironment and development, which may be more physiologically and medically significant, restricts our study design. Based on the available evidence, breast cancer EVs can target Y79 cells, proving that their molecular makeup is distinct. To understand the surface indicators responsible for the enhanced cellular absorption in Y79 cells, we would need to analyze the surface markers in detail using protein arrays or proteomics. Future studies might look at the effectiveness of 4T1 and SKBR3 Dox-loaded EVs in in vitro 3D models and in vivo retinoblastoma models to see if our findings hold true in more complex disease models. Furthermore, we cannot rule out the potential that Dox-loaded EVs have different effects on retinoblastoma in vivo than they did in vitro.

Overall, our findings contribute significantly to the developing concept that cancer-cell-derived EVs can be used as an efficient drug delivery tool for retinoblastoma treatment. We provide evidence that Dox-loaded breast-cancer-derived EVs may hold promise as a novel drug delivery candidate. When compared to free Dox, these Dox-loaded EVs had increased intracellular drug absorption and resulted in improved therapeutic effectiveness against retinoblastoma cells in vitro. EVs may offer a novel delivery tool for retinoblastoma and possibly other ocular diseases. More research is needed to determine the in vivo potential of Dox-loaded cancerous EVs in the management of retinoblastoma.

## 5. Conclusions

The current study provides evidence that EVs are a viable drug delivery technology due to their successful inherent cell-targeting capabilities and biocompatibility. We show that our generated Dox-loaded EVs display improved cellular internalization over free Dox in retinoblastoma cancer cells, resulting in enhanced cytotoxicity of the tumor cells. We found that Dox-loaded EVs enhanced caspase activation that is indicative of increased apoptosis. Our study demonstrated that there is increased antitumor activity of Dox-loaded EVs in targeting retinoblastoma cells in vitro, which may open new avenues for treating retinoblastoma with minimal side effects.

## Data Availability

Not applicable.
